# Single-Cell RNA Sequencing Uncovers Heterogeneous Circulating Tumor Cell Subsets in Breast Cancer

**DOI:** 10.3390/cancers14051314

**Published:** 2022-03-04

**Authors:** Maria A. Papadaki, Sofia Agelaki

**Affiliations:** 1 Laboratory of Translational Oncology, School of Medicine, University of Crete, 70013 Heraklion, Greece; papadaki_maria1@yahoo.gr; 2 Department of Medical Oncology, University General Hospital of Heraklion, 71110 Heraklion, Greece

## Introduction

Metastasis remains the main cause of death for breast cancer (BC) patients, and conceivably, a huge effort has been directed toward the understanding of the metastatic process [1]. Circulating tumor cells (CTCs), identified in the peripheral blood (PB) of patients with BC and other solid tumors, have been recognized as important precursors of metastasis [2]. Accordingly, their detection represents a valuable prognostic biomarker predicting poor survival measures in BC, and at the same time, it holds a promising role in monitoring disease progression [3]. Importantly, CTC analysis offers the unique opportunity to explore the biological characteristics of tumor cells that participate in the formation of metastases, thus expanding our knowledge on the mechanisms underlying disease progression [4]. Finally, CTC analysis can inform on the molecular characteristics of the tumor in real time, which could serve as an important tool for guiding personalized therapy in BC [5]. 

Recently, cutting-edge methodologies, such as RNA sequencing (RNA-seq) and single-cell RNA-seq (scRNA-seq), are increasingly being used to uncover the specific biological features of CTCs that drive metastatic progression and resistance to therapy. Specifically in BC, Boral D. et al. utilized whole-genome mRNA microarray and pathway analyses to show that CTCs from patients with documented brain metastases demonstrated enhanced activation of Notch signaling and increased pro-inflammatory chemokines, immunomodulatory networks, and mitogenic growth factors [6]. More recently, transcriptomic analysis of CTCs from triple-negative breast cancer (TNBC) patients revealed a liver metastasis-associated TNBC gene signature, which uncovered valuable prognostic and drug candidate biomarkers for TNBC [7]. scRNA-seq of CTCs has also been employed in other malignancies, such as multiple myeloma, hepatocellular, pancreatic, and prostate cancer, offering critical insights into the biology of metastasis and the mechanisms of resistance [8,9,10,11].

However, in most of these studies, RNA-seq analysis was restricted to CTCs selected according to their “classical” definition, which in general requires positivity for epithelial markers, such as cytokeratins (CKs) and epithelial-cell adhesion molecule (EpCAM), along with negativity for the common lymphocytic marker CD45 [12]. It should be noted here that this definition has been consistently used for the detection and enumeration of CTCs, providing significant prognostic information for patients with different types of cancer, including BC [2]. However, it is also widely accepted that CTCs constitute a highly heterogeneous cell population, which can frequently lose epithelial marker expression in the context of epithelial-to-mesenchymal transition (EMT) [13]. Especially in BC, a growing body of evidence supports the notion that CTCs bearing semi-EMT-like, or even fully mesenchymal-like phenotypes, also demonstrate cancer stem cell (CSC) properties and enhanced survival, immune evasion, and metastatic potential, as well as resistance to conventional therapies [6,14,15,16]. Thus, it is critical to appreciate that the analysis of the entire CTC compartment is critical to delineate CTC heterogeneity in order to uncover specific tumor characteristics that drive metastatic progression.

### The Study by Pauken M.C. et al., Published in Cancers

Pauken M.C. and co-authors [17], in an effort to describe the whole spectrum of CTC subsets that contribute to CTC heterogeneity and metastatic potency, utilized the transcriptomic analysis of white blood cells from 21 metastatic BC patients. Their experimental strategy included the isolation of cells based on lineage expression through flow cytometry, followed by analysis using the cutting-edge technologies of RNA-seq and scRNA-seq. Through the interrogation of both the Lineage− and Lineage+ (Lin−/Lin+) cells, the study revealed a “CTC-candidate” cell cluster harboring distinct gene expression patterns that subjects the classical definition of CTCs to revision and highlights the importance of exploring the properties of the entire population of CTCs in order to gain further insights into the putative mechanisms of metastasis.

More specifically, the initial RNA-seq analysis revealed a high number of differentially expressed genes (DEGs) between the Lin+ and Lin− cell populations. The Lin+ cell population was enriched with genes suggestive of an immune cell phenotype, such as PTPRC (CD45), CD3, CD4, CD8, CD27, and HEY1). Among the most significant Lin– DEGs identified were CAVIN2, ITGB3, LY6G6F, TUBB1, LTBP1, and TRIM58, as well as genes suggestive of an epithelial phenotype (e.g., EPCAM, TACSTD2, MUC2, KRT7, 8, 18, and 19), and genes specific to mammary tissue (e.g., LTF and CTTN). Further analysis of these genes revealed the enrichment of several pathways, including EMT, apical junctions, focal adhesion, estrogen response, and angiogenesis. These findings implied that the epithelial/mammary cells were enriched in the Lin– population.

To further delineate individual differences in gene expression between Lin− and Lin+ subsets, scRNA-seq analysis was indicatively performed in three BC patients who were selected based on high CTC counts (identified as epithelial-like CTCs using the RareCyte II platform) and had different hormone receptor status and metastases sites. The 10x Genomics Chromium clustering identified 15 distinct immune cell clusters, which were defined as T cells (five clusters), NK cells (two clusters), B cells (one cluster), macrophages (one cluster), neutrophils (two clusters), and monocytes (four clusters), with platelets being detected within most of these clusters. Importantly, the analysis revealed one cluster of cells with transcriptomic profiles not associated with immune cells, defined as “CTC-candidate” cluster, consisting of 201 cells from both the Lin– and the Lin+ population. This cluster was highly enriched with genes associated with epithelial cells and cell adhesion (e.g., KRT7, 9, 18, and 19, TBX3, TACSTD2, EPCAM, CLDN4, CLDN7, CEACAM6, and MGP), with EPCAM expression being unique to this cluster. It should be noted that many of these genes were expressed in less than 75% of cells within the cluster, further highlighting the significant degree of heterogeneity of CTCs in BC [18]. Interestingly, several Lin+ cells within the CTC-candidate cluster expressed CD45 either alone or in combination with epithelial markers (EPCAM, TACSTD2, CKs), which is in line with previous evidence that cells co-expressing CD45 and EpCAM or CKs can be identified in the PB of cancer patients [19].

Further analysis of RNA-seq data revealed that the “CTC-candidate” cluster was highly enriched with genes associated with cell adhesion, survival, and cell-to-cell communication. Specifically, genes encoding for claudins and tetraspanins, and other cell adhesion genes, such as MGP, PERP, RAB25, DSP, EMP2, PHLDA2, ERBB2, CEACAM6, and CTNND1, were extensively expressed in this cluster. Moreover, the cluster was enriched with genes coding for signaling proteins (e.g., CRABP2, CAMK2N1, PLK2, RHOV, SPINT1, RALA, and RERG) and proteins implicated in the response to calcium levels (TACSTD2, SRI, S100A16, S100A14, and CRACR2B) and to estrogens (CITED1, CITED4, GATA3, KRT19, CCND1, and IGFBP2). In addition, critical cell proliferation genes (CCND1, ANAPC11, and CDKN1A) and other genes with proliferative roles (TGFB1, ILK, BRK1, CIB1, AREG, and HES1) were also highly expressed. On the other hand, the immune cell clusters were highly enriched with genes associated with immune cell activation and inflammatory response, such as CCL5, IL32, and GZMK. Taken together, these findings imply that CTCs show enhanced expression of genes that activate survival signals and, in parallel, modulate their interactions with circulating immune cells. It is critical to note here that the mechanisms promoting the immune evasion capacities of CTCs and the tumor–immune crosstalk in the periphery represent a promising field in cancer research, with potential prognostic and therapeutic implications for BC patients [14,20,21,22,23].

The analysis was further focused on genes for transcription factors (TFs), oncogenes, and a series of genes associated with EMT and cancer stem cells (CSCs) within the “CTC-candidate” cluster. Interestingly, Lin– cells demonstrated more heterogeneous and patient-specific patterns, as compared to the Lin+ cells of the cluster. Thus, particular genes for TFs (e.g., LYL1, MAX, and TSC22D), as well as oncogenes (e.g., KIF5BTPM4 and NCOA4) were expressed in Lin− subpopulations, while, in contrast, Lin+ cells were clearly enriched with several TFs and oncogenes. Similarly, the Lin– populations presented a variable expression of epithelial genes (KRT7, 8, 18, and 19, CLDN 4 and 7, EPCAM, TACSTD2, ERBB3, ERBB2, JUP, and CD24) and a remarkably differential expression of several CSC genes, whereas Lin+ fractions were highly homogeneous, according to the expression of genes for mesenchymal-like and CSC phenotypes. These findings strongly imply that distinct cell populations co-exist within the CTC compartment, which could demonstrate different metastatic capacities and resistance or sensitivity to different therapies.

To summarize, Pauken M.C. et al. [17], performed a comprehensive characterization of CTCs in BC patients by employing an unbiased transcriptomic analysis of Lin– and Lin+ cells via scRNA-seq. Their approach allowed the identification of a “CTC-candidate” cluster with a distinct gene expression signature enriched with genes promoting cell adhesion, proliferation, cell-to-cell communication, as well as with genes for TFs, oncogenes, and genes that drive EMT and CSCs, which collectively may foster cell migration and metastatic dissemination. In addition, a significant heterogeneity at the single-cell level was demonstrated for the cells of the cluster. Importantly, the results of the current study question the “classical” CTC definition and point toward the identification of CTCs according to functionalities associated with metastatic potency, rather than according to the expression or absence of specific markers.

The success rate of CTC sequencing is limited by a series of technical issues, such as cell loss, damage of genetic material during isolation, leukocyte contamination, and difficulties in obtaining high quality of sequencing libraries [9,10]. Thus, CTC sequencing approaches cannot, as yet, be incorporated into routine CTC assessment, however, they are largely used in translational research for the recognition of biological parameters and pathways specific to metastasis-competent and therapy-resistant CTCs. Although circulating tumor DNA (ctDNA) sequencing is technically less challenging and offers significant information on the genomic and epigenomic landscape of the tumor [24,25], however CTCs can be additionally analyzed at the transcriptome level to uncover DEGs and key regulatory pathways that are active at a given time, thus providing a broad view of the phenotype and dynamics of the tumor [26]. Furthermore, CTC sequencing at the single-cell level can unmask the underlying heterogeneity of the tumor within each individual patient [27,28]. Herein, Pauken M.C. et al., in line with a limited number of other studies, underline the importance of performing transcriptomic analyses of CTCs isolated by an epithelial marker independent strategy [7,11,29,30]. A further important step should be to delineate the biological function of the CTC subsets identified by Pauken M.C. et al., to explore whether their different transcriptomic patterns are in fact associated with differences in their metastatic potential. In addition, considering that these results were obtained by analyzing a limited number of patients, it is crucial to investigate the distribution of these CTC subpopulations in a larger cohort of BC patients and their associations with patient outcomes.

To conclude, based on this and other reports [7,31] it is likely that the transcriptomic analysis of CTCs will serve in the near future for the discovery of novel prognostic signatures and of therapeutic strategies that may enhance the implementation of precision medicine and will improve the clinical management of BC.
